# Correlation between C9ORF72 mutation and neurodegenerative diseases: A comprehensive review of the literature

**DOI:** 10.7150/ijms.53550

**Published:** 2021-01-01

**Authors:** Xingfeng Xu, Yan Su, Zhenyou Zou, Yali Zhou, Jianguo Yan

**Affiliations:** 1 Faculty of Basic Medical Sciences, Guilin Medical University, Guilin, 541004, Guangxi, China.; 2 Guangxi Key Laboratory of Brain and Cognitive Neuroscience, Guilin Medical University, Guilin, 541004, Guangxi, China.

**Keywords:** C9ORF72, Neurodegenerative diseases, Amyotrophic lateral sclerosis, Frontotemporal dementia, Alzheimer's disease, Parkinson's disease

## Abstract

Chromosome 9 open reading frame 72 (C9ORF72) encodes a 54-kDa protein with unknown function that is expressed at high levels in the central nervous system. The C9ORF72 hexanucleotide amplification is one of the most recently discovered repetitive amplification diseases related to neurodegeneration. Its association with amyotrophic lateral sclerosis/frontotemporal dementia (ALS/FTD) spectrum diseases has been fully established, although a causative role for C9ORF72 in Alzheimer's disease (AD) and Parkinson's disease (PD) remains to be established. Therefore, in this article, we will review the evidence for C9ORF72 as a causative factor in neurodegenerative diseases, the underlying mechanisms, and the potential for targeting C9ORF72 as a strategy to alleviate neurodegenerative disease progression.

## Introduction

Neurodegenerative diseases, characterized by the massive loss of specific neurons, are complex, progressive, disabling, and often fatal conditions that can be divided into acute and chronic neurodegenerative diseases. The former mainly include stroke and brain injury, while the latter mainly include amyotrophic lateral sclerosis (ALS), Parkinson's disease (PD), and Alzheimer's disease (AD). Although the lesions and causes of these diseases are different, degenerative neuronal lesions are common to both classes. Neurodegeneration is the gradual loss of neuronal structure and function through mechanisms that include neuronal death and glial cell imbalance, which can cause forms of cognitive impairment, such as dementia. To date, no strategies to slow the progression of neurodegenerative diseases have been identified. This lack of therapeutic options is due, in large part, to our incomplete understanding of the molecular and cellular mechanisms that cause neurodegeneration.

The chromosome 9 open reading frame 72 (C9ORF72) gene is located on the short arm of chromosome 9 (9p21) and encodes 11 exons. The hexanucleotide GGGGCC repeat of the C9ORF72gene is located in intron 1 of the non-coding region and is located between exons 1a and 1b. This hexanucleotide segment can occur only once or be repeated multiple times in a row; estimates suggest repeats of up to 30 have no negative effect on gene function. The C9ORF72 gene encodes a protein that is found in various tissues. In the nervous system, the C9ORF72 protein is expressed in all regions of the brain, with the highest expression level in the cerebellum. The protein is abundant in nerve cells (neurons) in the outer layers of the brain (cerebral cortex) and in specialized neurons in the brain and spinal cord that control movement (motor neurons). The C9ORF72 protein is thought to affects the process of gene transcription, RNA translation into protein, and the transport of RNA within the cell.

Through a complex process of alternative splicing, three C9ORF72 transcripts are produced. Transcript 1 and transcript 3 have the same transcription start site, whereas the transcription start site of transcript 2 is upstream of the pathogenic GGGGCC repeat mutation sequence. These three transcripts are translated to produce two protein subtypes. Transcript 1 produces the shorter sequence C9ORF72 protein subtype 1, consisting of 222 amino acids, while transcripts 2 and 3 produce the same longer C9ORF72 protein subtype 2, consisting of 481 amino acids. Quantitative mRNA expression analysis indicated that the GGGGCC repeat expansion abolished the expression of the C9ORF72 transcript 1 from the mutant allele. When the C9ORF72 gene is mutated, transcripts 1 and 3 can generate pathogenic dipeptide repeat (DPR) aggregates. Therefore, understanding how C9ORF72 leads to neurodegenerative diseases is important for the identification of treatments for neurodegenerative diseases. Our knowledge is increasing regarding the role and underlying mechanism of C9ORF72 in the pathogenesis of various neurodegenerative diseases, such as amyotrophic lateral sclerosis (ALS), frontotemporal dementia (FTD), Parkinson's disease (PD), and Alzheimer's disease (AD).

## The relationship between C9ORF72 and ALS/FTD

ALS is a chronic and progressive neurodegenerative disease. It is the most common type of motor neuron disease, which manifests as progressively worsening skeletal muscle weakness and atrophy. The age of onset is mainly between 30 and 60 years, affecting more men than women. FTD is a sporadic or inherited dementia involving the frontal and temporal lobes of the brain. It is a chronic, systemic condition that is usually manifested as irreversible cognitive decline. FTD accounts for 10% of all cases of dementia. The age of onset is usually younger than that for AD (55-65 years), and the incidence is the same for men and women. C9ORF72 hexanucleotide amplification is a newly discovered repetitive amplification disease related to neurodegeneration. Its association with FTD/ALS spectrum diseases has been well established and is considered to be one of the leading related genes. Preliminary evidence shows that, in patients with clinically diagnosed ALS in China, Japan, South Korea, and Taiwan, the C9ORF72 mutation rate is much lower than that observed in the Caucasian population, which indicates that the number of repeats varies greatly depending on nationalities and ethnicities 1-4.

### How mutated C9ORF72 leads to ALS/FTD

With continuous advances in clinical, pathological and basic research, and the development of gene sequencing technology, many studies have shown overlapping pathogenic genes, pathological characteristics and clinical manifestations of ALS and FTD. ALS and FTD are pathologically and genetically linked neurodegenerative diseases that share common dysregulated molecular pathways 5,6. ALS causes progressive loss of motor neurons and muscle weakness 7, whereas FTD causes focal atrophy in the frontal and temporal lobes, resulting in progressive cognitive and behavioral alterations and other deficits 8. Basic research has shown that FTD and ALS have similar pathogenic mechanisms 9; therefore, FTD and ALS are classified as a FTD-ALS spectrum disease 10. GGGGCC hexanucleotide repeat expansion (HRE) in the first intron of C9ORF72 is the most common genetic cause of both ALS and FTD 11,12. The GGGGCC HRE in the C9ORF72 gene is found in as much as 40% of cases of familial ALS and FTD 11-13.

#### Formation of nuclear RNA foci

A RNA fluorescence *in situ* hybridization (FISH) study using a probe targeting the GGGGCC repeat sequence (probe (GGCCCC)_6_) conducted in paraffin-embedded sections of postmortem tissues revealed multiple RNA lesions in 25% of the nuclei in the frontal cortex and spinal cord of patients with FTLD-TDP (FTLD-TDP is a subtype of frontotemporal dementia with genetic heterogeneity. Neuropathological examination shows TDP43-positive inclusion bodies, and 20% of them have GRN (human telomerase RNA gene) mutation expression positive. However, no foci were observed in any of the samples when using a probe targeting the unrelated CCTG repeat (probe (CAGG)_6_), further supporting that the RNA lesion is composed of GGGGCC 7. In another study, treatment of induced pluripotent stem cell (iPSCs) with RNase A and DNase I confirmed the specificity of the FISH probe. RNase A and DNase I reduced and maintained RNA foci, respectively, which strongly suggests that these nuclear inclusions are composed of “GGGGCC” RNA rather than DNA 14.

Paraspeckles are nuclear ribonuclear bodies, an essential paraspeckle proteins that is colocalizes with the longer non-coding RNA (lncRNA) NEAT1. Paraspeckles are involved in regulating the post-transcriptional process of cells by chelating RNA binding protein (RBP), mRNA and microRNA and their nuclear retention and subcellular/subnuclear compartmentalization. NEAT1_2 has been shown to be essential for the formation of paraspeckle, it provides a speckle assembly framework sufficient to maintain the integrity of the paraspeckles. Studies have shown that (GGGGCC) n RNA foci and paraspeckles are similar in structure and function. First, the researchers showed that (GGGGCC) n RNA foci (pathological features associated with C9ORF72) are colocalizes with the essential paraspeckle proteins SFPQ, NONO, RBM14, hnRNPH and FUS, similar to the structure of paraspeckles. The hypothetical paraspeckle function is sequestration of RBPs or RNA molecules. Therefore, upregulating the amount of NEAT1_2 leads to larger paraspeckles and therefore sequestration of more paraspeckles-related proteins from the nucleoplasm, which leads to reduced availability. A similar phenomenon can be caused by potential (GGGGCC)_n_ RNA foci in ALS/FTD pathology associated with C9ORF72. Second, the upregulation of NEAT1 in the brain of FTD patients, and the possibility of formation of alternative paraspeckle-like foci by (GGGGCC)_n_ RNA increase the importance of paraspeckle and paraspeckle-like structures in the pathogenesis of ALS and FTD. Finally, it was also observed that (GGGGCC)_72_ RNA foci formed paraspeckle-like bodies in a NEAT1-independent manner. This finding suggests that (GGGGCC)_n_ RNA repeats can replace the NEAT1-RNA as a scaffold in a paraspeckle-like structures.

#### C9ORF72 HRE-induced cytotoxicity

It has been suggested that the cytotoxicity induced by the C9ORF72 HRE is caused by loss and gain of function. These mechanisms include: 1) Transcription of sense GGGGCC_exp_ or antisense (CCCCGG_exp_) RNA, which sequester proteins to change their normal function 11. The pathogenesis of noncoding repeat-amplified diseases may be due to the accumulation of RNA transcripts that contain repeated amplification, which mediate isolation of RNA binding proteins (RBPs) 14. The presence of RNA foci in C9ORF72 ALS cells suggests that the amplified GGGGCC RNA may also isolate RBPs. Previous studies have revealed that the ADARB2 protein is colocalized with the nuclear GGGGCC RNA foci, and the ADARB2 protein interacts with the C9ORF72 RNA with high binding affinity for the GGGGCC repeat RNA sequence both biochemically and in live cells. Furthermore, these *in vitro* observations were reproduced *in vivo*. Evaluation of the possible requirement of ADARB_2_ for RNA foci formation indicated that the interaction between ADARB_2_ and C9ORF72 RNA amplification plays a role in the formation or maintenance of RNA foci *in vitro*, thus providing evidence in support of the involvement of the interaction of RBPs and GGGGCC repeat sequences in GGGGCC_exp_ RNA toxicity 15. 2) Translation of the sense or antisense expanded RNA by repeating the relevant non-AUG translation to form a toxic dipeptide repeat protein (DPR) 16-19. Five DPR proteins - poly(GP), poly(GA), poly(PA), poly(GR), and poly(PR) - are found in the brains of patients with C9ORF72-ALS/FTD 16,17,20. Among these proteins, poly(GR) expression is closely related to neurodegeneration in C9ORF72-ALS/FTD 21. Although the underlying mechanism has not been fully understood, poly(GR) expression itself has been shown to cause neurotoxicity both *in vitro* and *in vivo* 18,19,22. It has also been demonstrated that HRE RNA forms hairpin and G-quadruplex structures that bind and sequester RBPs 15,23.

#### Insoluble polypeptides specific to C9FTD/ALS

Given that the abnormal accumulation of insoluble proteins in many neurodegenerative diseases is related to neuronal dysfunction and degeneration, the identification of disease-specific C9RANT-positive pathology in patients with C9ORF72 repeat expansion may provide valuable insights for disease mechanisms and potential treatment strategies. Not only can the abnormal aggregation of RAN translation products be targeted, but also RNA structures that are considered essential for RAN translation can be formed. As a new discovery in the specific neuropathology of C9FTD/ALS, the accumulation of poly-(glycine-proline) peptides provides a new perspective for the pathological biology of C9FTD/ALS. Although it remains to be determined whether the polypeptide produced by RAN translation is neurotoxic. The RNA structure of the GGGGCC repetitive sequence makes these transcripts susceptible to non-traditional translation mechanisms, namely repeat-related non-ATG (RAN) translation. The initiation of RAN translation is believed to depend on the RNA hairpin structure using C:G complementary pairing. Therefore, GGGGCC RNA transcripts are likely to form stable hairpins and therefore may undergo RAN translation. To confirm that poly-(glycine-proline) peptides are specifically expressed in C9FTD/ALS, synthetic peptides (GA)_8_, (GP)_8_ and (GR)_8_ were used as antigens to immunize two rabbits and prepared two independent polyclonal antibodies (designated anti-C9RANT) with high specificity and sensitivity for the hypothetical RAN translation product. The antibodies were used to show the existence of C9RANT immunoreactive inclusion bodies in the central nervous system (CNS) of C9FTD/ALS patients, and they were found only in gray matter and neurons. No C9RANT immunoreactive lesions were found in other neurodegenerative lesions. Anti-C9RANT antibodies are specific for C9FTD/ALS CNS neurological contents. Furthermore, C9RANT-positive inclusion bodies are associated with a high molecular weight insoluble substance. The development of anti-C9RANT immunoassay technology has facilitated studies of the C9RANT immune response in cerebrospinal fluid as a potential marker of disease activity and/or progression.

#### The C9ORF72 repeat expansion disrupts nucleocytoplasmic transport

Recent research has indicated that repeated amplification of GGGGCC disrupts nuclear and cytoplasmic transport in drosophila models and human cells. The NPC and nucleocytoplasmic transport defects observed in iPSC-derived neurons and motor neurons were found to be associated with ALS and FTD. That is, the C9ORF72 HRE product disrupts the nucleocytoplasmic transport of NPC and is the basic mechanism by which cell damage is induced in ALS/FTD. The high levels of cytoplasmic Ran GTP prevents the formation of the NLS-introduced protein αβ complex, thereby disrupting the N/C Ran gradient and damaging the nuclear import of NLS-containing proteins 24.

### Targeting the C9ORF72 gene mutation to treat ALS/FTD

#### RNA toxicity is mitigated by antisense intervention

Using the ALS-iPSCs system, it was shown that all toxic phenotypes were alleviated by treatment of induced pluripotent stem cell-derived neurons (iPSNs) with novel antisense oligonucleotides (ASOs) targeting GGGGCC_exp_ RNA sequences, while C9ORF72 RNA levels were not reduced. This new antisense therapy can be used to treat neurodegeneration caused by repeated amplification of C9ORF72 15.

#### RanGAP, a key regulator of nucleocytoplasmic transport, as a potent suppressor of neurodegeneration

GGGGCC RNA protein interactors, including RanGAP_1_, have been identified in recent studies. RanGAP (Drosophila orthologous gene of human RanGAP_1_) is an effective inhibitor of neurodegenerative changes 24. RanGAP in the cytoplasm stimulates Ran-GTPase to hydrolyze GTP, resulting in the generation of GDP, which is critical for effective nuclear and cytoplasmic transport processes 25-27. In C9-ALS, GGGGCC HRE binds and sequesters RanGAP_1_, resulting in an increase in cytoplasmic Ran GTP 24. Overexpression of nuclear Ran guanine nucleotide exchange factor (RanGEF) enhances GGGGCC repeat-mediated degeneration. In contrast, knockout of the RanGEF gene rescues this degeneration. Increasing nuclear import or inhibiting nuclear export of proteins can also inhibit neurodegenerative changes. This degeneration can be prevented by importin α overexpression or exportin knockout. Therefore, gene-enhanced nuclear import or suppression of nuclear localization sequence (NLS) and/or nuclear export signal (NES) protein output inhibits GGGGCC-mediated neurodegeneration.

#### AFF_2_/FMR_2_ regulates the transcription and toxicity of the amplified GGGGCC repeat sequence in human C9ALS/FTD neurons

CRISPR-Cas9-mediated loss of AFF_2_/FMR_2_ (one of four human homologs of Lilli) specifically reduces the levels of C9ORF72 RNA, RNA foci, and DPR protein in iPSC-derived neurons from C9ORF72-ALS/FTD patients, and can rescue axonal degeneration and TDP-43 pathology 21. Using CRISPR-Cas9 technology to conduct unbiased genetic screening of neurons derived from C9ORF72-induced pluripotent stem cells (iPSC), a Drosophila poly(GR) model was established. In this model, poly(GR) expression in adult neurons resulted in axonal and motor defects and premature death without obvious TDP-43 pathology. To further investigate how AFF_2_ regulates C9ORF72 expression in human neurons, CRISPR-Cas9 technology was used to knock out AFF_2_ in an iPSC line derived from two C9ORF72 patients. AFF2 knockout significantly reduced the level of C9ORF72-variation 3, while isotype variation 2 or antisense RNA did not, and AFF_2_ preferentially regulated the transcription of C9ORF72 alleles containing extended GGGGCC repeats in patient neurons. Thus, partial knockout of AFF2 may represent a potential treatment.

#### Strategies aimed at diminishing the function of SUPT_4_H_1_ could be pursued as an effective therapy for C9FTD/ALS

The specific expression of hexanucleotide repeat amplification in the C9ORF72 gene was found to require the transcription factor Spt_4_/SUPT_4_H_1_ (Spt_4_ is a highly conserved transcription elongation factor, and SUPT_4_H_1_ is a human Spt_4_ homologous gene) 28. Single-factor targeted therapy has a greater advantage in eliminating the pathological characteristics of CFTD/ALS than targeting the sense and antisense repeats separately. Targeting the transcription elongation factor Spt4 to reduce the expression of a single gene product, SUPT_4_H_1_ or SUPT_5_H, can reduce the three main pathological features of C9FTD/ALS - sense RNA foci, antisense RNA foci, sense and antisense extended transcripts and their translated dipeptide repeats (DPR) products - and reduce degradation in animal models. Spt_4_ deletion specifically and significantly reduces the level of poly(GP) translated by RAN caused by the extended nucleotide repeat sequence, but does not inhibit the toxicity of three non-repetitive disease-associated proteins [TDP43, FUS/TLS, Poly(PR)]. In the C9ORF72 repeat amplification vector, the mRNA expression levels of SUPT4H1 and SUPT5H are positively correlated with the levels of C9ORF72 variant 3 mRNA or poly (GP) protein. Furthermore, related studies showed that knocking out SUPT_4_H_1_ affects extended repeat transcription, but has little effect on other the transcription of other sequences.

## The relationship between C9ORF72 and AD

AD is the most common progressive neurodegenerative disease with occult attacks 29. Clinically, it is characterized by general symptoms of dementia, such as memory loss, aphasia, apraxia, dementia, impaired visuospatial skills, executive dysfunction, and personality and behavioral changes. However, the pathology of AD remains to be clarified. Due to the overlap in the clinical and pathological features of the common neurodegenerative diseases FTD, ALS and AD, and considering the clinical heterogeneity of repeated sequence expansion, we hypothesize that the length of the repeated sequence may also be related to AD. Indeed, studies have shown that C9ORF72 is expressed in dystrophic neurites that accumulate on senile plaques in the AD brain 30.

To study the hexanucleotide repeat amplification of C9ORF72 in different genetic backgrounds, Jiao et al. screened C9ORF72 in large groups of PD (n = 911), AD (n = 279) and ET (n = 152) patients in the Chinese Han population using the repeated-start polymerase chain reaction method. However, no pathogen duplications (>30 duplications) were detected in the patients or the control group (n = 314), indicating that C9ORF72 may have little pathogenic expansion in these three diseases 31. These findings support the latest data from other independent studies. The first possibility is that hexanucleotide repeat amplification does not cause PD, AD or ET, and are specific only to ALS/FTD. Another explanation is that the threshold for ALS/FTD repeats >30 units is not sufficient to for detection in AD, PD, or ET patients, and more samples should be included to set a reliable threshold. In this study, a positive correlation was observed between the I/I genotype (I, allele [≥7 units]) and the age of onset, which indicates that AD patients with the I/I genotype are prone to older onset 31. It is speculated that the pathogenic repeat expansion is most likely to be related to advanced AD (>65 years).

Currently, APOE_4_ is the only major genetic risk factor for the development of late-onset AD 32. If this relationship is further verified, then future research will focus on exploring the relationship between C9ORF72 repeat sequence and the APOE genotype in patients with late-onset AD. Li et al. studied the relationship between C9ORF72 repeat sequence amplification (≥30 repeat sequences), intermediate repeat sequence copies (20-29 repeat sequences) and AD by searching the PubMed, EMBASE and Cochrane databases. After a rigorous screening process, 10 articles were identified for the required final statistical analysis. The limited number of original articles about the association between the intermediate duplicate copies of C9ORF72 and AD affected the validity of the analysis. There was no obvious heterogeneity in the results included in the meta-analysis. The results showed that C9ORF72 repeat expansion was associated with AD risk (OR = 6.36, 95% CI = 3.13-12.92, *P* <0.00001), while C9ORF72 intermediate duplicate copies were not (OR = 1.04, 95% CI = 0.32-3.43, *P* = 0.94) 33.

Amnestic frontotemporal lobar degeneration (FTLD) may be misdiagnosed as AD, or the verbal form of AD can be interpreted as progressive non-fluent aphasia (PNFA). Therefore, another study investigated whether the most common genetic cause of FTLD (i.e., repeat expansion in C9ORF72) is present in a large number of clinical diagnoses of AD. The number of hexanucleotide repeats in all tested AD patients was within the normal range of control subjects (1-23 repeats). This study included only one patient, and the maximum number of repeats identified in the AD cohort was 21. These findings indicated that this particular mutation has nothing to do with amnestic FTLD. Therefore, the C9ORF72 extension seems to be specific to FTLD and ALS, rather than the cause of AD 34.

Kaivola and other researchers studied the length of C9ORF72 hexanucleotide repeats in 3,161 individuals from four populations in Helsinki, southern Finland. From the four cohorts, 226 AD patients, 432 MMSE 24 patients and 613 patients diagnosed with dementia or cognitive impairment were identified. The repeat length was successfully determined in 3,142 of 3161 patients (99.4%) by them. In 12 individuals (0.38%) with 30-45 repeats, no obvious neurodegenerative diseases or psychiatric clusters were identified, indicating that 30-45 repeats are not pathogenic 35. Of the 3,142 cases, only six were repeat expansion carriers (>45 repeats) and all were from the largest (HBCS) cohort. Six expansion carriers were not diagnosed with ALS/FTD, while four were diagnosed with neurodegenerative or psychiatric disease. These data were consistent with studies showing that medium-length alleles (classified as 7-45 and 20-45 duplications) are not related to AD or cognitive impairment, supporting the hypothesis that seven duplication alleles do not cause the damage that leads to cognitive impairment or dementia 35.

However, another study showed that the role of intermediate repeat copies is closely related to neurodegenerative diseases and C9ORF72 expression. With the increase in the number of normal repeats, the transcriptional activity of C9ORF72 decreased significantly, indicating that intermediate repeat copies may act as susceptible alleles and promote the disease via a loss of function mechanism 36. An *in vitro* study showed that the transcriptional activity of the C9ORF72 promoter decreased significantly with the increase from normal to intermediate repeats. Compared with the two units on the wild-type allele, the transcriptional level of the promoter containing 24 units was decreased by more than 50% 36. Another study showed that in human renal neuroblastoma cells (HEK293T cells), intermediate repeats (7-24) were associated with significantly lower promoter activity than normal short repeats (2-6 units) 37. These findings indicate that increasing the length of the repeat sequence can lead to a decrease in promoter activity, which is due to the increase in methylation of the CpG sequence in the larger repeat sequence and transcriptional silencing of the promoter 37.

To confirm the role of C9ORF72, Cacace et al. evaluated the incidence of GGGGCC pathogenic repeat expansion in patients from Flanders in Belgium with clinical AD or mild cognitive impairment (MCI). In the study cohort, all pathogenic GGGGCC repeats carried the T allele in rs2814707. However, according to analysis of the association with GGGGCC repeat length, there was no significant relationship between rs2814707 and AD 38. It is worth noting that the frequency of GGGGCC repeat amplification in the study cohort was comparable to that in the previous study cohort (<1%) 39. This confirms that C9ORF72 GGGGCC repeat expansion is not a common cause of AD, but may still be the basis of the neurodegenerative process, with a clinical phenotype compatible with AD 40. In the autopsy series described by Murray and colleagues, most of the C9ORF72 mutation carriers who showed symptoms of MCI or AD amnesia had hippocampal sclerosis 41. In the study by Cacace et al., one of the patients had a typical amnestic MCI, with significant bilateral hippocampal volume reduction. Hippocampal sclerosis may mimic the clinical manifestations of AD by affecting memory dysfunction, leading to an initial diagnosis of AD. The brain anatomy of patients undergoing GGGGCC repeat amplification was clearly diagnosed as AD, which further confirms that the existence of C9ORF72 GGGGCC repeat amplification in the AD patient cohort cannot be fully explained by misdiagnosis as FTLD 39.Therefore, it can be concluded that the pathogenic C9ORF72 GGGGCC repeat expansion can be detected in clinical AD patients, and may be the cause of the pathogenesis of AD, although C9ORF72 is not a direct AD risk factor 38.

## The relationship between C9ORF72 and PD

PD is a common degenerative disease of the nervous system. It is more common in the elderly and the average age of onset is about 60 years. The prevalence of PD in people aged over 65 years in China is approximately 1.7%. PD is rare in people under the age of 40 years. Most patients with PD are sporadic, and less than 10% of patients have a family history. The most important pathological change in PD is the degeneration and death of dopaminergic neurons in the substantia nigra of the midbrain, which causes a significant decrease in dopamine (DA) content in the striatum, which causes disease.

In many studies, C9ORF72 accounts for a small fraction typical PD cases. Atypical Parkinson's syndromes also exist, including chronic brain syndrome (CBS), progressive supranuclear palsy (PSP) and multiple system atrophy (MSA). Features that increase the possibility of a positive test include early cognitive and/or behavioral symptoms, a positive family history of ALS or FTD, and the presence of UMN (upper motor neuron) and LMN (lower motor neuron) signs. More and more reports indicate that C9ORF72 expansion may be related to a series of other phenotypes, including neuropsychiatric and dementia manifestations, movement disorders and other complex neurodegenerative syndromes 42,43. However, there are, as yet, no reports clarifying the specific association between C9ORF72 expansion and PD or other movement disorders, and its clinical significance outside the FTD/ALS spectrum. Only a few studies have reported other symptoms, mainly Parkinsonism and psychotic features, in several C9ORF72-positive FTD/ALS patients 44-46. The incidence of Parkinsonism and PD is also increased in C9ORF72 amplified ALS/FTD patients 44 and their relatives 44,47,48. A study showed that the prevalence of C9ORF72 expansion is high (>20%) among PD patients who exhibit the clinical symptoms and meet the clinical criteria for the diagnosis of ALS 49. Another study showed that in patients with a behavioral variant FTD (bvFTD) phenotype, the prevalence of PD symptoms reached 50% to 75%, which is much higher than that in patients with the ALS phenotype 44,50.

To clarify the role of (GGGGCC)n expansion in PD, Theuns et al. evaluated the C9ORF72 (GGGGCC)n repeat sequence in the GEO-PD (Genetic Epidemiology of Parkinson's Disease) cohort, which includes patients from Europe, Asia, North America and Australia. Only four PD patients were identified with pathogenic (GGGGCC)n expansion (>60), and all four had a positive family history of neurodegenerative dementia, ALS or atypical PD. No carriers were found to have typical sporadic or familial PD. The meta-analysis showed that the risk of PD increased with the number of (GGGGCC)n repeats; however, no strong correlation between C9ORF72 (GGGGCC)n repeats and PD was detected. Calculation of the risk of each observed C9ORF72 (GGGGCC)n allele in the GEO-PD cohort suggested that the combined allele of 10 units and 17 or more units plays a role in PD susceptibility. However, none of the associations were retained after Bonferroni correction, suggesting that even if the C9ORF72 repeat sequence of 10 units or more plays a role in PD susceptibility, the effect is small 51. In addition, in a cohort of sporadic and familial PD phenotypes, five patients were found to have extensive expansion, although none of the C9ORF72-positive patients had a typical family history of PD 52. Taken together, these findings indicate that the C9ORF72 expansion has no major role in the pathogenesis of PD.

A large number of patients with C9ORF72 (GGGGCC)>60 extended FTLD/ALS developed atypical PD in the early stage of the disease, and the incidence of PD increased among relatives with or without complex features of FTLD/ALS 10,11,53. These data support the current hypothesis that the pathogenicity (GGGGCC)n repeat amplification in C9orf72 is specific to the FTLD/ALS profile. Therefore, C9ORF72 (GGGGCC)n repeat amplification tests are not recommended for patients with typical PD who are diagnosed based on medical genetics. Nevertheless, there can be exceptions for PD patients with cognitive and/or behavioral deficits in the early stages of the disease process or patients with a personal or family history of FTLD/ALS. To determine the histopathological basis of the incidence of PD observed in families with a history of ALS/FTD with C9ORF72 expansion, Johnathan et al. extracted DNA from 377 brains with a histopathological diagnosis of idiopathic PD (iPD) or related diseases, and analyzed the amplification of C9ORF72. Of the 338 cases that were pathologically confirmed as iPD, only one had C9ORF72 expansion. Compared with the ALS brain without C9ORF72 expansion, the loss of dopaminergic neurons in the substantia nigra (SN) of the ALS brain with C9ORF72 expansion is more obvious 54. It has been noted clinically 55 and neuropathologically 47,56 that the SN is involved in amyotrophic lateral sclerosis (ALS). Immunohistochemical analysis of α-synuclein and p62 expression in the SN of 17 cases of brains with C9ORF72 expansion and 51 cases of ALS without C9ORF72 expansion, showed that only one case had α-synuclein-positive neuron cell pulp content (with C9ORF72 expansion) 54. Alpha-synuclein pathology does not exist in the SN of the vast majority of C9ORF72 expanded brains, which further strengthens our hypothesis that the intracellular mechanism that causes neuronal cell loss in ALS/FTD is different from the mechanism that causes iPD to cause α-synuclein pathology. In contrast, the incidence of p62-positive and TDP-43-negative neuronal cytoplasmic inclusions in SN is much higher in brain tissues with C9ORF72 expansion than in brains without C9ORF72 expansion. Therefore, this p62-positive extrapyramidal pathology may account for the previously reported C9ORF72-related ALS-related features of PD 54.

In previous reports, the frequency of the PD phenotype in families 13,47 and incidental cases 44,46,47 with C9ORF72 expansion is higher than that in those with the ALS/FTD phenotype without C9ORF72 expansion 54. Therefore, it seems that the intron expansion of C9ORF72 at least partially explains the observed association between ALS and PD. However, it is not clear whether carriers of C9ORF72 expansion mutations develop Parkinson's syndrome (as observed in iPD) due to α-synucleinopathy caused by C9ORF72 expansion, or whether the underlying pathology of these patients is more in line with the typical C9ORF72 expanded extra-motor pathology (p62 positive, TDP-43 negative, ubiquitinated neurons and glial cytoplasmic inclusion bodies) 47. It is worth noting that so far, the 9p21 locus has not been included in iPD genetic association studies 57. Until the pathogenesis of C9ORF72 disease is fully understood, it is still impossible to rule out C9ORF72 amplification as a rare cause of α-synucleinopathy and clinical iPD. One study demonstrated that intermediate amplification of the C9ORF72 gene (>20 duplicate copies) increases the risk of PD and essential tremor plus Parkinson's disease (ETP) 58. These researchers used repeated primer PCR assays to determine the repeat size of a total of 889 clinically confirmed patients (including PD and ETP) and 1,144 controls. It was found that amplification of large C9ORF72 repeats (>30 repeats) did not increase the risk of PD 58. However, compared with the control, the intermediate duplication (>20 to 30+) of PD and ETP cases increased significantly. There were 14 cases (13 PD, 1 ETP) and three controls with >20 duplicate copies, seven cases and none of the controls had >23 duplicate copies. In addition, in a small number of families in this study, the intermediate RC allele lacked isolation from clinical PD, and intermediate/extended repeat sequences were detected in some controls. This suggests that the intermediate RC allele is most likely a risk factor for susceptibility rather than having a causal relationship with PD/Parkinsonism 58. Taken together, these data indicate that the C9ORF72 repeat sequence leads to a significant risk of PD/Parkinsonism.

## Conclusion

At present, C9ORF72 is known to cause ALS/FTD, and there have been some reports describing methods of intervention for C9orf72. In contrast, because there are few reports discussing the relationship between C9orf72 and AD, the impact of C9ORF72 on AD is still unclear, with no studies that clearly indicate whether C9ORF72 is related to this condition. However, detection of the pathogenic C9ORF72 GGGGCC repeat expansion in clinical AD patients indicates that C9ORF72 may be the cause of AD, rather than a direct risk factor. For PD, the most common argument is that PD is bundled with ALS/FTD, and the incidence of PD will increase in people with a family history of ALS/FTD. Studies have also indicated that intermediate amplification of the C9ORF72 gene (>20 duplicate copies) increases the risk of PD and essential tremor plus PD (ETP), although the specific mechanism requires further exploration.

## Abbreviations

C9orf72: Chromosome 9 open reading frame 72
ALS: Amyotrophic lateral sclerosis
FTD: Frontotemporal dementia
AD: Alzheimer's disease
PD: Parkinson's disease
DPR: Dipeptide repeat
SN: Substantia nigra
NLS: Nuclear localization sequence
NES: Nuclear export signal
